# Optimization of Butterworth and Bessel Filter Parameters with Improved Tree-Seed Algorithm

**DOI:** 10.3390/biomimetics8070540

**Published:** 2023-11-11

**Authors:** Mehmet Beşkirli, Mustafa Servet Kiran

**Affiliations:** 1 Department of Computer Engineering, Karamanoğlu Mehmetbey University, 70200 Karaman, Türkiye; 2 Department of Computer Engineering, Konya Technical University, 42250 Konya, Türkiye

**Keywords:** tree seed algorithm (TSA), Butterworth filter, Bessel filter, Parameter extraction, optimization

## Abstract

Filters are electrical circuits or networks that filter out unwanted signals. In these circuits, signals are permeable in a certain frequency range. Attenuation occurs in signals outside this frequency range. There are two types of filters: passive and active. Active filters consist of passive and active components, including transistors and operational amplifiers, but also require a power supply. In contrast, passive filters only consist of resistors and capacitors. Therefore, active filters are capable of generating signal gain and possess the benefit of high-input and low-output impedance. In order for active filters to be more functional, the parameters of the resistors and capacitors in the circuit must be at optimum values. Therefore, the active filter is discussed in this study. In this study, the tree seed algorithm (TSA), a plant-based optimization algorithm, is used to optimize the parameters of filters with tenth-order Butterworth and Bessel topology. In order to improve the performance of the TSA for filter parameter optimization, opposition-based learning (OBL) is added to TSA to form an improved TSA (I-TSA). The results obtained are compared with both basic TSA and some algorithms. The experimental results show that the I-TSA method is applicable to this problem by performing a successful prediction process.

## 1. Introduction

Optimization techniques are used to solve real-world problems that are difficult or complex to solve [1]. Optimization means reaching the best possible result by using the available possibilities [2]. Meta-heuristic algorithms, which are generally inspired by nature, are used in the literature to solve engineering problems that are often complex [3]. Optimal solutions of the solved problems are estimated based on the methods used [4]. According to the No Free Lunch theory, there is no single algorithm for solving optimization problems [5]. For this reason, there are many meta-heuristic algorithms in the literature for optimum solutions to real-world engineering problems [6,7,8]. In this study, parameter adjustment was the aim for Butterworth and Bessel active filters, which is one of the real-world problems. A filter is a device that passes electrical signals at certain frequencies or frequency ranges while blocking the passage of others. Jiang et al. [9] proposed the clonal selection algorithm for selecting the optimal components for the Butterworth filter design. It has been said that the experimental results are much more successful than the studies in the literature. Shakoor et al. [10] presented a genetic algorithm-based optimization approach for the parameter optimization of active filters. They said that it can be used to design all kinds of active filters according to the analysis results. De et al. [11] proposed a particle swarm optimization algorithm for the design of active filters. According to the test results, it is stated that the design error of active filters is minimized thanks to this proposed method. Temurtas [12] made some improvements to the charged system search algorithm (CSS). Both the CSS and the proposed method are used to determine the parameters of the Butterworth filter design problem. Doğan and Ölmez [13] proposed the vortex search algorithm for the component selection of active filters. When the results are compared with the results obtained using algorithms, such as particle swarm optimization, artificial bee colony, the differential evolution algorithm and harmony search algorithm, it is said that the proposed algorithm finds the optimum values for both active filter topologies. Durmuş et al. [14] improved the differential evolution algorithm. Their proposed method is applied to the Chebyshev filter design problem. Optimum parameter values, filter components, and quality factor values are given. It is stated that the proposed method will be a reference for future studies by achieving success based on this problem. Vural et al. [15] used the differential evolution algorithm and harmony search algorithm from metaheuristic algorithms for optimum filter design. Experimental results indicated that both the differential evolution algorithm and the harmony search algorithm minimize the total design error in active filtering. In this study, the tree seed algorithm (TSA), which is inspired by nature, is proposed for parameter optimization of the Sallen–Key topology high- and low-pass active Butterworth and Bessel filters. When the literature is examined, it is seen that the TSA has been applied to many problems. Beşkirli and Dağ [16] used the original TSA to extract the parameters of STM6-40/36 solar panels using three different models. It is said that the TSA performs well on this problem. Ghaouti and Meftah [17] conducted a study on brain MR images. Since the brain has a complex structure, it is said that MR images are not homogeneous. For this reason, they used the TSA to segment and cluster MR images. They stated that the TSA obtained better and satisfactory results compared to the fuzzy c-mean algorithm in this experimental study using a real data set. More [18] optimized the discrete wavelet transform approach in the image constraint technique by using the TSA. It is stated that the algorithm has its own techniques and thus achieves success. Beşkirli and Dağ [19] provided information about the performance of TSA by using the original TSA in the inference of solar panel parameters, such as RTC France, PWP201, using three different models. Mandal et al. [20] aimed to obtain minimum values for the autonomy days (ADs), system cost efficiency, loss of power supply probability (LPSP) and renewable franchise (RF) of a microgrid operating with a renewable energy system. They performed these operations by making some improvements to the TSA. According to the results they obtained, they stated that they achieved noticeable success in terms of the percentage with the proposed algorithm. Venkatasubramanyan et al. [21] used optimization techniques to reduce energy consumption and extend the network lifetime in WSNs. While performing these operations, they proposed a more effective hybrid method by adding the tree seed algorithm (TSA) in order to use the moth search algorithm more effectively. Here, the effect of TSA is stated to be the multipath routing process. They said that the proposed method provides good performance in multipath routing. Liu et al. [22] improved the performance of TSA by adding some improvements to improve the performance of the TSA. They used their proposed method to solve the test functions. According to the results obtained here, they stated that the proposed method has good performance. Sharshir et al. [23] used an artificial neural network (ANN) to estimate the amount of fresh water produced by a conventional and improved solar distillation method. They aimed to improve the performance of the ANN by using the TSA for the estimation of the weights in the ANN. According to their results, they concluded that ANN is a better prediction tool thanks to the TSA. Jiang et al. [24] stated that they improved the performance of TSA by making some improvements to the TSA. To demonstrate this, they solved test functions and engineering problems with the proposed method. They stated that the results they obtained were successful and added that the proposed method has become a promising candidate. Lui et al. [25] stated that the performance of the TSA increased as a result of the improvements made in the update mechanism of the TSA. In order to prove this, they stated that they obtained successful results by solving engineering problems, as well as test functions, with the proposed method. They emphasized that the proposed method is important in terms of feasibility and effectiveness. In this study, the TSA was improved by the OBL method and applied to the filter design problem. The improved TSA was named I-TSA. I-TSA was compared with both the basic TSA and the results of PSO and CSS algorithms. In addition, I-TSA and TSA were analyzed separately according to the ST parameters. Convergence plots, box plots and gain plots, obtained as a result of all of these analyses, are presented in the relevant sections.

The following sections of the study are designed as follows. In Section 2, the TSA method is explained. In Section 3, the improved TSA method is explained. In Section 4, the design of the filters is mentioned. In Section 5, the experimental results obtained according to the filter types are given. In the last chapter (Section 6), the conclusion and suggestions are given.

## 2. Tree Seed Algorithm

**Figure 1:**
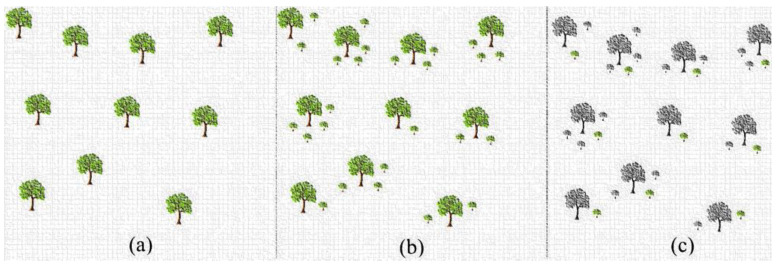
Working diagram of basic TSA (**a**) Initialization (**b**) Seed production (**c**) Seed selection.

The TSA, inspired by nature, was proposed by Kıran in 2015 [26]. The TSA is formed from the interaction of the positions of trees and seeds in the search space. The best tree in the population or randomly selected tree position is used for each seed production. The most important parameter of the TSA method is the ST control parameter. This parameter ensures the diversity of seed production. This diversity is realized using the formulas in Equations (1) and (2) [27]. Here, if the randomly selected number is less than the ST parameter value, the first equation is used, and if it is larger, the second equation is used.

(1) $$S_{i,j}=T_{i,j}+\alpha_{i,j}\times \left(B_{j}-T_{r,j}\right)$$

(2) $$S_{i,j}=T_{i,j}+\alpha_{i,j}\times \left(T_{i,j}-T_{r,j}\right)$$

where $S_{i,j}$ refers to the seeds produced. $T_{i,j}$ refers to the tree of the specified size. $\alpha_{i,j}$ is a random number generated between −1 and 1. $B_{j}$ denotes the best tree. $T_{r,j}$ denotes a tree randomly selected from the population. At the beginning of the search space, the initial population (tree locations), which is specified as possible solutions in optimization problems, is obtained using Equation (3) [28].

(3) $$T_{i,j}=L_{j,min}+r_{i,j}(H_{j,max}-L_{j,min})$$

where $L_{j,min}$ and $H_{j,max}$ are the lower and upper bounds of the search space, respectively. $r_{i,j}$ is a randomly generated value between 0 and 1. To select the best solution from the population, a function f is defined, which is used in Equation (4) [29].

(4) $$B=minf\left({\overset{\to }{T}}_{i}\right)\;\;\;\;\;\;\;i=1,2,\ldots ,N$$

where N stands for the trees in the population. The working diagram of the TSA is given in Figure 1 [30]. Here, trees are first planted in the search space (a), then seed production is performed for each tree (b), and finally seed selection is performed (c). The pseudo code of the TSA is given in Algorithm 1 [31].
**Algorithm 1** TSA pseudo-code**Step 1:** The initialization of the algorithm Randomly generate tree locations on the D-dimensional search space using Equation (3). Evaluate the tree locations by the fitness function. Select the best location using Equation (4). **Step 2:** Search with seeds **FOR** all trees  Decide the number of seeds produced for this tree.  **FOR** all seeds   **FOR** all dimensions    **IF** (rand < ST)     Update this dimension using Equation (1).     **ELSE**     Update this dimension using Equation (2).     **END IF**   **END FOR**  **END FOR**  Select the best seed and compare it with the tree.  If the seed location is better than the tree location, the seed substitutes for this tree. **END FOR** **Step 3:** Selection of the best solution Select the best solution of the population. If new best solution is better than the previous best solution, new best solution substi-tutes for the previous best solution. **Step 4:** Testing the termination condition

## 3. Improved Tree Seed Algorithm

Since the initial population is randomly distributed in the search space of the TSA, initial solutions are generated randomly. Instead, by using the opposition-based learning (OBL) method [32], the TSA can obtain a better initial population and by continuing the process, the TSA can produce successful solutions. The OBL method was first introduced in 2005 and has been used in many studies. Thus, the OBL method aims to improve the performance of the algorithm. In this study, the OBL method evaluates the solution of the problem while at the same time generating an opposing solution so that the TSA will have the chance to produce a solution closer to the global optimum in the search space [33]. According to the OBL method, when a and b are real numbers, the value of x is calculated as in Equation (5).

(5) $$\overset{⌣}{x}=a+b-x$$

## 4. Filter Design Problem

A filter is a device that passes electrical signals in a certain frequency range without undergoing any change and prevents signals from other frequencies from passing [34]. There are two types of filter designs. The first is the passive filter, and the second is the active filter. Passive filter design incorporates resistors, capacitors and coils as circuit elements, whereas semiconductor circuit elements, like transistors, are added to the circuit along with passive circuit elements for active filter design. In this study, low-pass active Butterworth and Bessel filters (LPAFs) and high-pass active Butterworth and Bessel filters (HPAFs) were designed by connecting five filters in succession and increasing to the 10th order in Sallen–Key topology, which is an electronic filter topology with a second-order active filter. In this study, resistors suitable for the E24 series were used. All nominal resistance values of the E24 series resistors are given in Table 1. The E24 series comprises preferred resistor values enabling electronic component designers and manufacturers to select from a practical range of values. This set was developed to simplify the design process.

### 4.1. Design and Equations of LPAF

**Figure 2:**
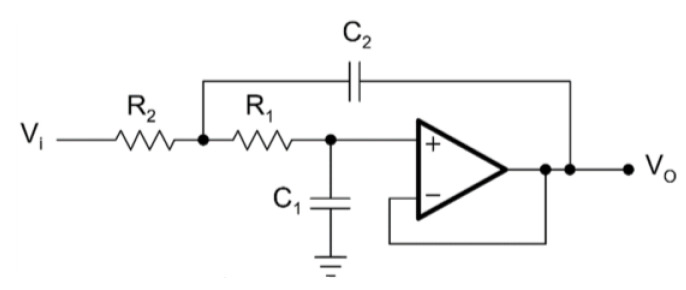
Second-order LPAF.

**Figure 3:**
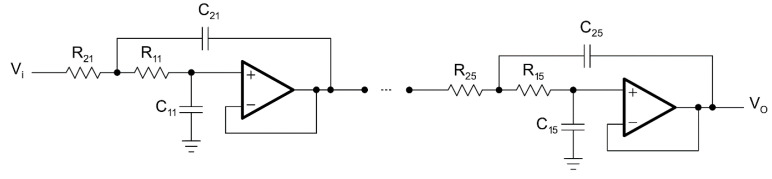
Tenth-order LPAF.

The second order in Sallen–Key topology and the tenth order in Sallen–Key topology circuit diagrams for the LPAF are given in Figure 2 and Figure 3, respectively.

The equations for the transfer function of the circuit, standard form and amplitude effect are given in Equations (6)–(8), respectively [12,35,36,37,38].

(6) $$\begin{array}{c} H_{LPF}(s)=\frac{V_{o}(s)}{V_{i}(s)}=\frac{1}{1+s(R_{1}+R_{2})C_{1}+s^{2}R_{1}R_{2}C_{1}C_{2}},s=j2\pi f\\ H_{LPF}(f)=\frac{1}{1-{\left(2\pi f\right)}^{2}R_{1}R_{2}C_{1}C_{2}+j2\pi f(R_{1}+R_{2})C_{1}} \end{array}$$

(7) $$\begin{array}{c} H_{LPF}(f)=\frac{1}{1-{\left(\frac{f}{FSF\cdot f_{c}}\right)}^{2}+j\frac{f}{Q\cdot FSF\cdot f_{c}}}\\ FSF=\frac{1}{2\pi f_{c}\sqrt{R_{1}R_{2}C_{1}C_{2}}};Q=\frac{\sqrt{R_{1}R_{2}C_{1}C_{2}}}{(R_{1}+R_{2})C_{1}} \end{array}$$

(8) $$H_{LPF}(f)=\frac{1}{\sqrt{{\left(1-{\left(\frac{f}{FSF\cdot f_{c}}\right)}^{2}\right)}^{2}+{\left(\frac{f}{Q\cdot FSF\cdot f_{c}}\right)}^{2}}}$$

### 4.2. Design and Equations of HPAF

**Figure 4:**
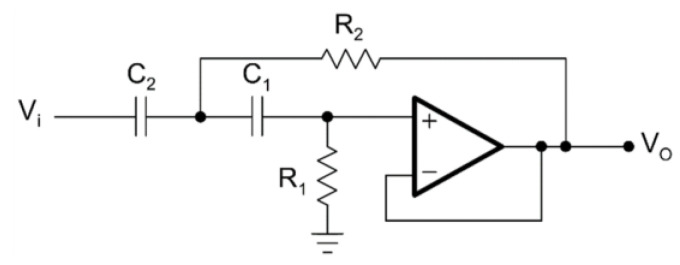
Second-order HPAF.

**Figure 5:**
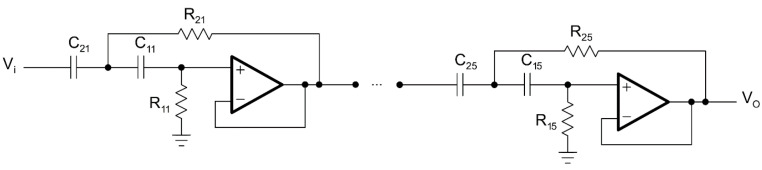
Tenth-order HPAF.

The second order in Sallen–Key topology and tenth order in Sallen–Key topology circuit diagrams for the HPAF are given in Figure 4 and Figure 5, respectively.

The equations for the transfer function of the circuit, standard form and amplitude effect are given in Equations (9)–(11), respectively [12,35,36,37,38].

(9) $$\begin{array}{c} H_{HPF}(s)=\frac{V_{o}(s)}{V_{i}(s)}=\frac{1}{1+\frac{C_{1}+C_{2}}{sR_{1}C_{1}C_{2}}+\frac{1}{s^{2}R_{1}R_{2}C_{1}C_{2}}},s=j2\pi f\\ H_{HPF}(f)=\frac{1}{1-{\left(\frac{1}{2\pi f\sqrt{R_{1}R_{2}C_{1}C_{2}}}\right)}^{2}-j\frac{C_{1}+C_{2}}{2\pi fR_{1}C_{1}C_{2}}} \end{array}$$

(10) $$\begin{array}{c} H_{HPF}(f)=\frac{1}{1-{\left(\frac{f_{c}}{FSF\cdot f}\right)}^{2}-j\frac{f_{c}}{Q\cdot FSF\cdot f}}\\ FSF=2\pi f_{c}\sqrt{R_{1}R_{2}C_{1}C_{2}};Q=\frac{\sqrt{R_{1}R_{2}C_{1}C_{2}}}{\left(R_{2}(C_{1}+C_{2}\right)} \end{array}$$

(11) $$\left|H_{HPF}(f)\right|=\frac{1}{\sqrt{{\left(1-{\left(\frac{f_{c}}{FSF\times f}\right)}^{2}\right)}^{2}+{\left(\frac{f_{c}}{Q\times FSF\times f}\right)}^{2}}}$$

### 4.3. Cost Function Errors

The total error calculation of the designed filter was calculated using Equations (12) and (13). This formula is obtained by summing the cost function errors of *FSF* values and *Q* values.

(12) $${Error}_{1}=\sum_{i=1}^{5}\frac{\left|{FSF}_{t,i}-{FSF}_{i}\right|}{{FSF}_{t,i}},\;{Error}_{2}=\sum_{i=1}^{5}\frac{\left|Q_{t,i}-Q_{i}\right|}{Q_{t,i}},$$

(13) $${Error}_{Total}=0.5\times {Error}_{1}+0.5\times {Error}_{2}$$

## 5. Experimental Results

The proposed I-TSA was used to estimate the parameters of the Butterworth and Bessel filter problem. Parameter optimization was also performed on the ST parameter of the I-TSA method, and the ST parameter was determined as 0.1, 0.5 and 0.9, respectively. The estimation results obtained for both Butterworth and Bessel filters are given in the tables, and the gain graphs are shown in the figures. The performance of the proposed I-TSA was also compared with the results of both the basic TSA and PSO and CSS algorithms. Convergence and box plots are also presented. All results are presented under the relevant headings.

### 5.1. Butterworth Filter (BWF) Results

#### 5.1.1. LPAF

The results obtained by applying the I-TSA method to the LPAF problem are given in this section. The values obtained as a result of the least error value obtained using the I-TSA method are given in Table 2. R1 (kΩ), R2 (kΩ), C1 (nF) and C2 (nF) values for each stage are shown in Table 2.

In Table 3, the FSF values and Q values estimated using the I-TSA method are given. It has been observed that the results obtained with the proposed method are very close to the targeted results. Especially when the ST value is 0.1, it has achieved a better performance in terms of FSF and Q values.

In Table 4, the parameter of the LPAF problem is the error value obtained as a result of the estimation with the I-TSA method. For the LPAF problem, the I-TSA, TSA, PSO and CSS was run 30 times, and the best, mean, worst and standard deviation values obtained are given in the table. When the table is examined, it is seen that the I-TSA method has the least error value when the ST value is 0.1. Therefore, it can be said that the parameter values in cases where the ST value is 0.1 are robust.

**Figure 6:**
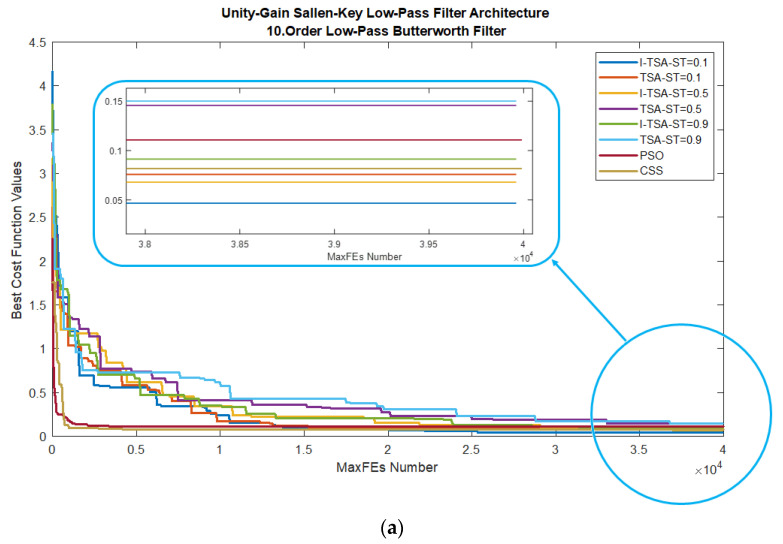

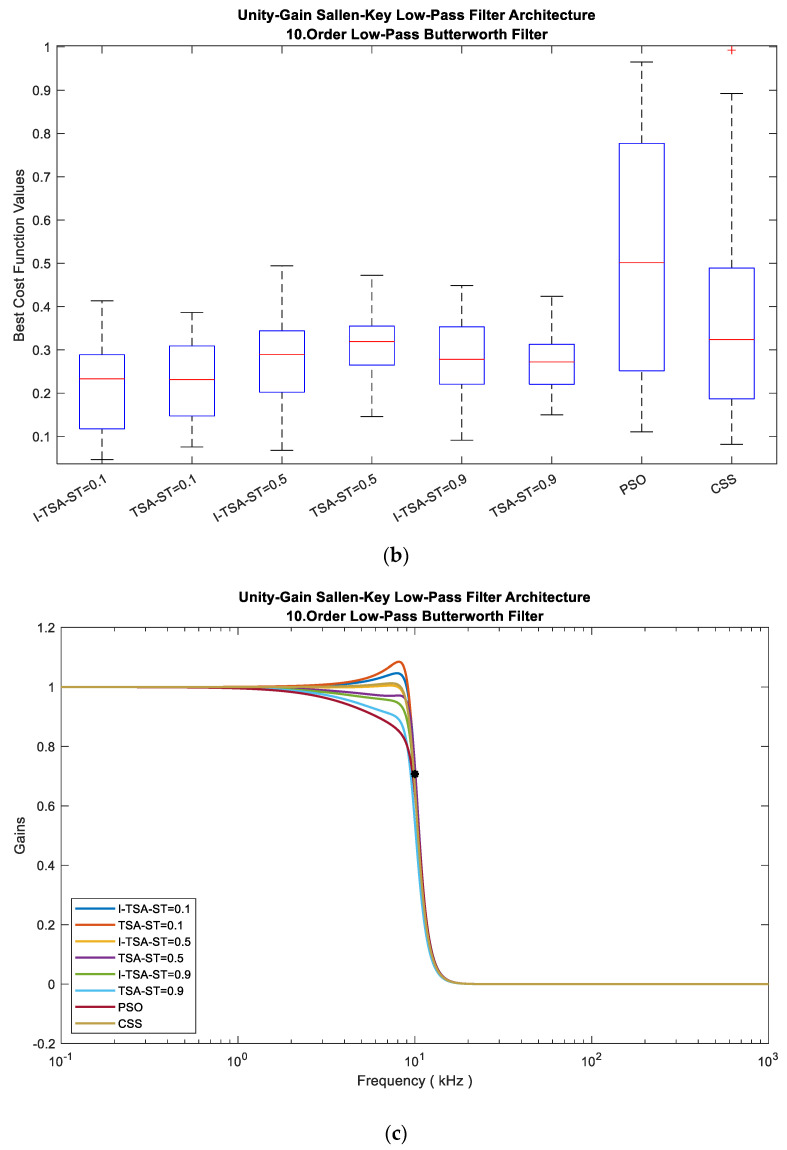

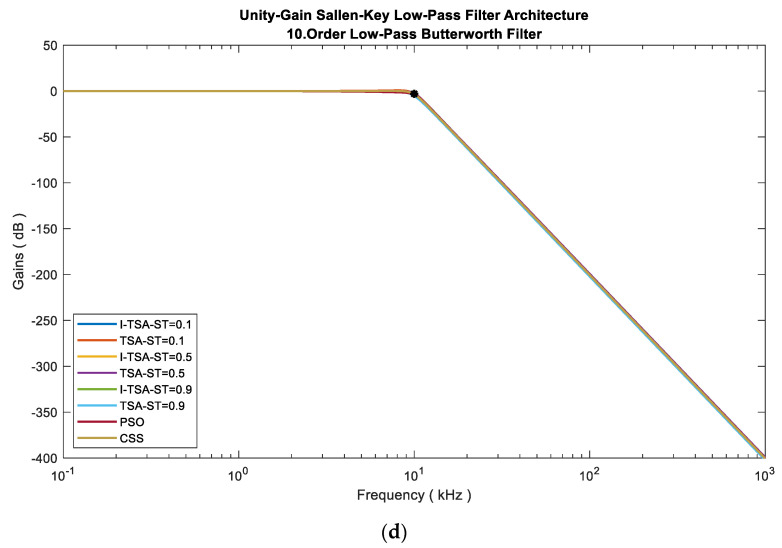
Plots for LPAF in the BWF: (**a**) convergence graphs of I–TSA, TSA, PSO and CSS; (**b**) box plots of I-TSA, TSA, PSO and CSS; (**c**) frequency and gain graphs of I-TSA, TSA, PSO and CSS; (**d**) frequency and gain (dB) graphs of I-TSA, TSA, PSO and CSS.

When analyzed according to all ST conditions of the I-TSA, TSA, PSO and CSS, the graphs in Figure 6 are obtained. In Figure 6a, it is seen in the MaxFEs graph that when the ST value is 0.1, I-TSA achieves a more stable convergence and reaches the minimum error value. The box plots obtained from here are given in Figure 6b. In addition, the gain and gain (dB) graphs based on the minimum error value according to the ST values are given in Figure 6c,d, respectively.

#### 5.1.2. HPAF

The results obtained by applying the I-TSA method to the HPAF problem are given in this section. The values of the minimum error value result obtained according to the ST values of the I-TSA method are given in Table 5. R1, R2, C1 and C2 values for each stage are shown in Table 5.

In Table 6, the FSF values and Q values estimated using the I-TSA method are given. It has been observed that the results obtained with the proposed method are very close to the targeted results. The closest value in terms of FSF and Q values was obtained with an ST value of 0.1. It was observed that the ST value of 0.1 was closer to the FSF and Q values.

In Table 7, the parameter of the HPAF problem is the error value obtained as a result of the estimation with the I-TSA method. For the HPAF problem, the I-TSA, TSA, PSO and CSS method was run 30 times, and the best, mean, worst, and standard deviation values obtained are given in the table. When the table is examined, it is seen that the I-TSA method has the least error value when the ST value is 0.1. Therefore, it can be said that the parameter values in cases where the ST value is 0.1 are robust.

**Figure 7:**
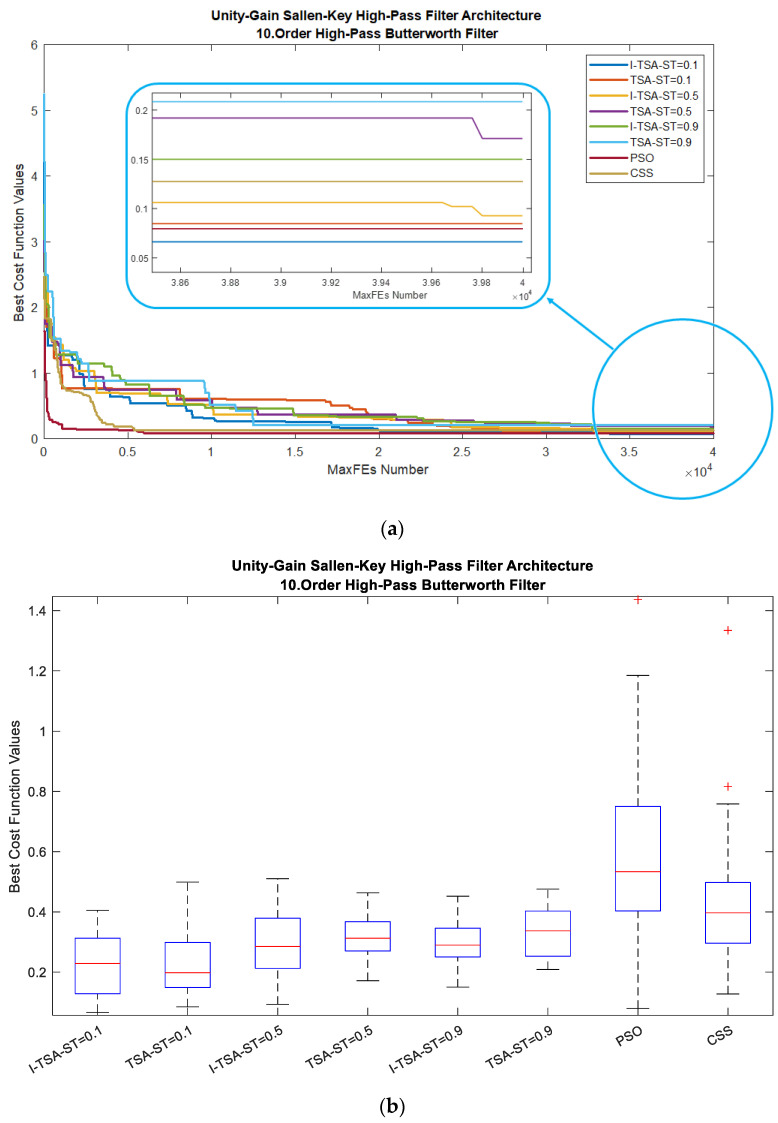

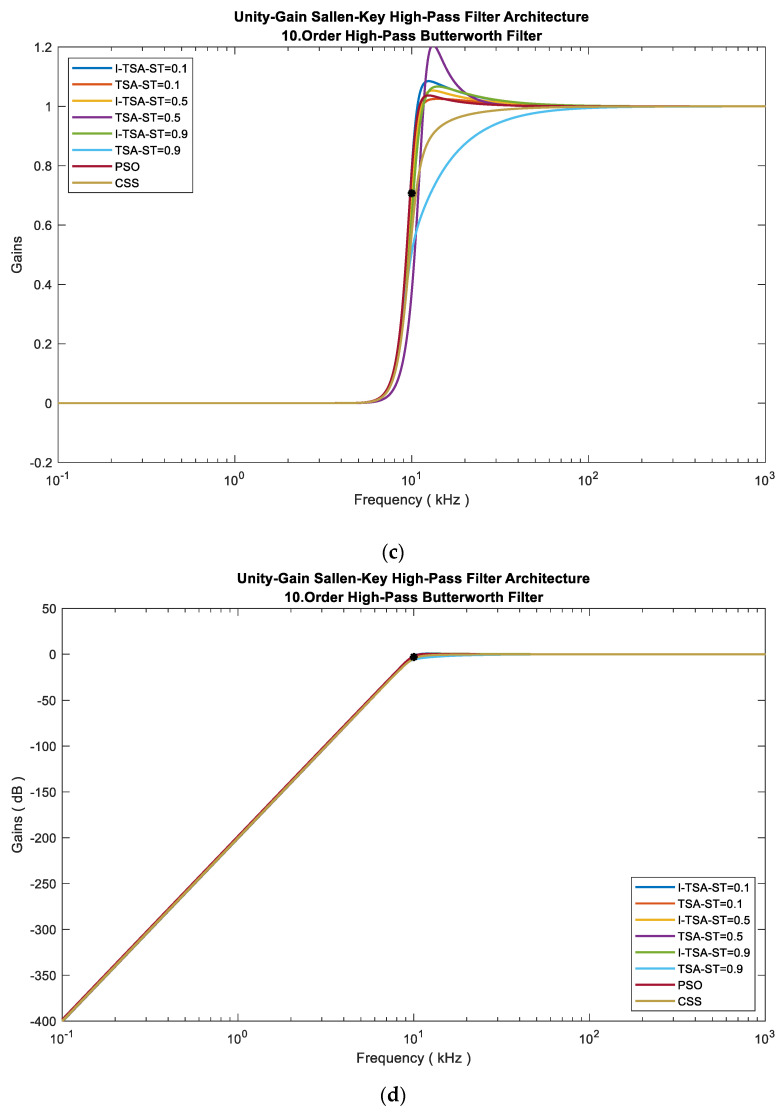
Plots for HPAF in the BWF: (**a**) convergence graphs of I-TSA, TSA, PSO and CSS; (**b**) box plots of I-TSA, TSA, PSO and CSS; (**c**) frequency and gain graphs of I-TSA, TSA, PSO and CSS; (**d**) frequency and gain (dB) graphs of I-TSA, TSA, PSO and CSS.

When analyzed according to all algorithms, the graphs in Figure 7 are obtained. In Figure 7a, it is seen in the MaxFEs graph that when the ST value is 0.1, I-TSA achieves a more stable convergence and reaches the minimum error value. The box plots obtained from here are given in Figure 7b. In addition, the gain and gain (dB) graphs based on the minimum error value according to the ST values are given in Figure 7c,d, respectively.

### 5.2. Bessel Filter (BF) Results

#### 5.2.1. LPAF

The results obtained by applying the I-TSA method to the LPAF problem are given in this section. The values obtained because of the least error value obtained by the I-TSA method are given in Table 8. R1, R2, C1 and C2 values for each stage are shown in Table 8.

Table 9 shows the FSF values and Q values estimated using the I-TSA method. It is seen that the results obtained with the proposed method are very close to the targeted results. The closest value in terms of the FSF value was obtained when the ST value was 0.1. The closest value in terms of the Q value was obtained when the ST value was 0.1.

In Table 10, the parameter of the LPAF problem is the error value obtained as a result of the estimation with the I-TSA, TSA, PSO and CSS method. For the LPAF problem, the I-TSA, TSA, PSO and CSS method was run 30 times, and the standard deviation values obtained are given in the table. When the table is examined, it is seen that the I-TSA method has the least error value when the ST value is 0.1. Therefore, it can be said that the parameter values in cases where the ST value is 0.1 are robust.

**Figure 8:**
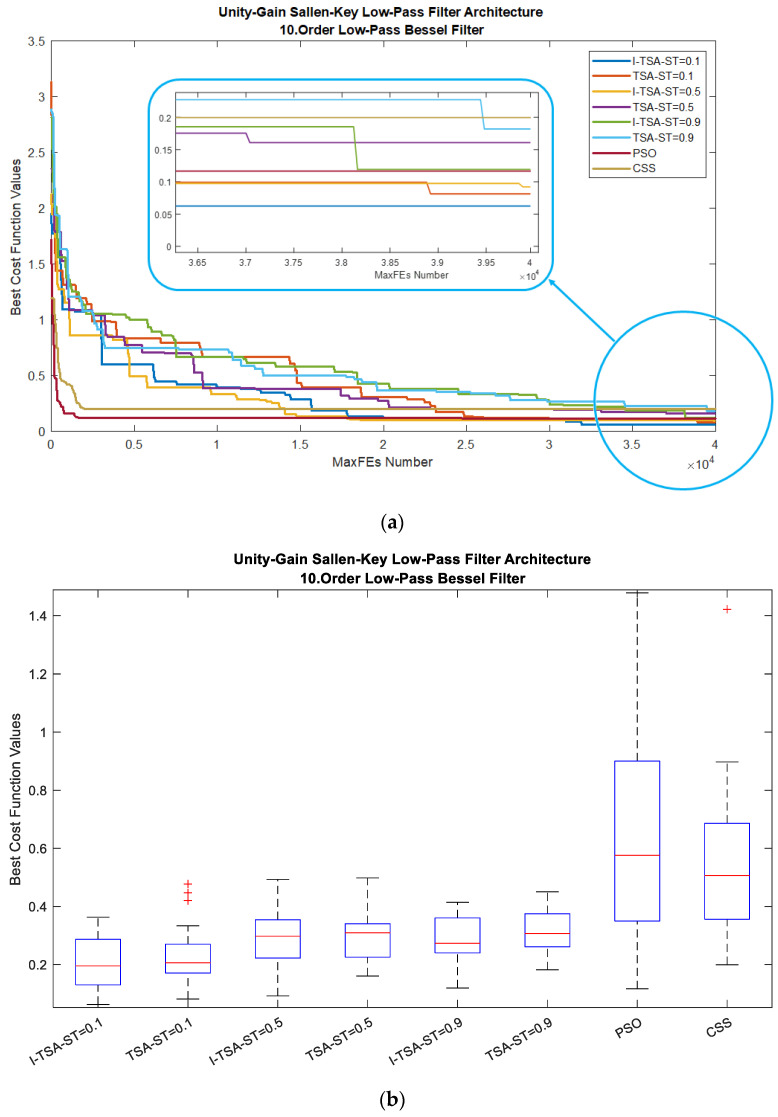

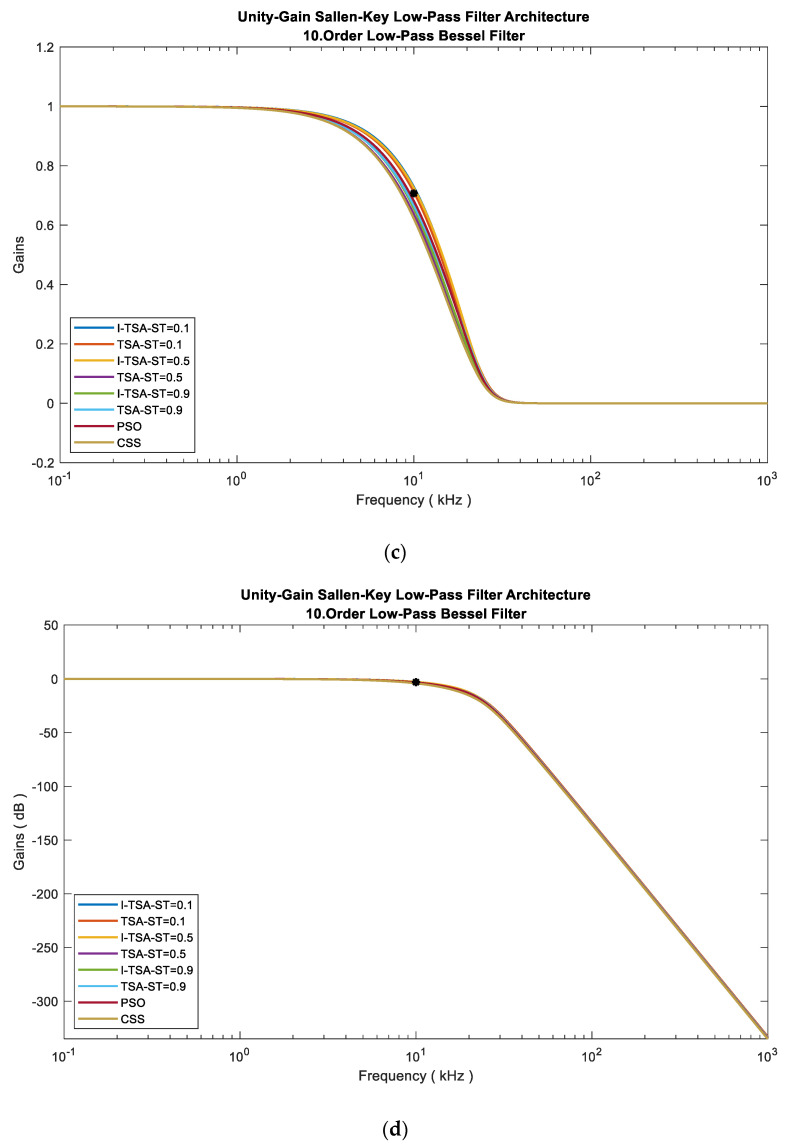
Plots for LPAF in the BF: (**a**) convergence graphs of I-TSA, TSA, PSO and CSS; (**b**) box plots of I-TSA, TSA, PSO and CSS; (**c**) frequency and gain graphs of I-TSA, TSA, PSO and CSS; (**d**) frequency and gain (dB) graphs of I-TSA, TSA, PSO and CSS.

When analyzed according to all algorithms, the graphs in Figure 8 are obtained. In Figure 8a, it is seen in the MaxFEs graph that when the ST value is 0.1, I-TSA achieves a more stable convergence and reaches the minimum error value. The box plots obtained from here are given in Figure 8b. In addition, the gain and gain (dB) graphs based on the minimum error value according to the ST values are given in Figure 8c,d, respectively.

#### 5.2.2. HPAF

The results obtained by applying the I-TSA method to the HPAF problem are given in this section. R1, R2, C1 and C2 values for each stage are shown in Table 11, the FSF and Q values are given in Table 12 and the finally, a comparison among the swarm intelligence algorithms have been given in in Table 13. 

Table 12 shows the FSF values and Q values estimated using the I-TSA method. It is seen that the results obtained with the proposed method are very close to the targeted results. The closest value in terms of the FSF value was obtained when the ST value was 0.5 and 0.9. In terms of the Q value, the closest value was obtained when the ST value was both 0.1 and 0.9.

In Table 13, the parameter of the HPAF problem is the error value obtained as a result of the estimation with the I-TSA method. For the HPAF problem, the I-TSA, TSA, PSO and CSS method was run 30 times, and the standard deviation values obtained are given in the table. When the table is examined, it is seen that the TSA method has the least error value when the ST value is 0.1. Therefore, it can be said that the parameter values in cases where the ST value is 0.1 are robust.

**Figure 9:**
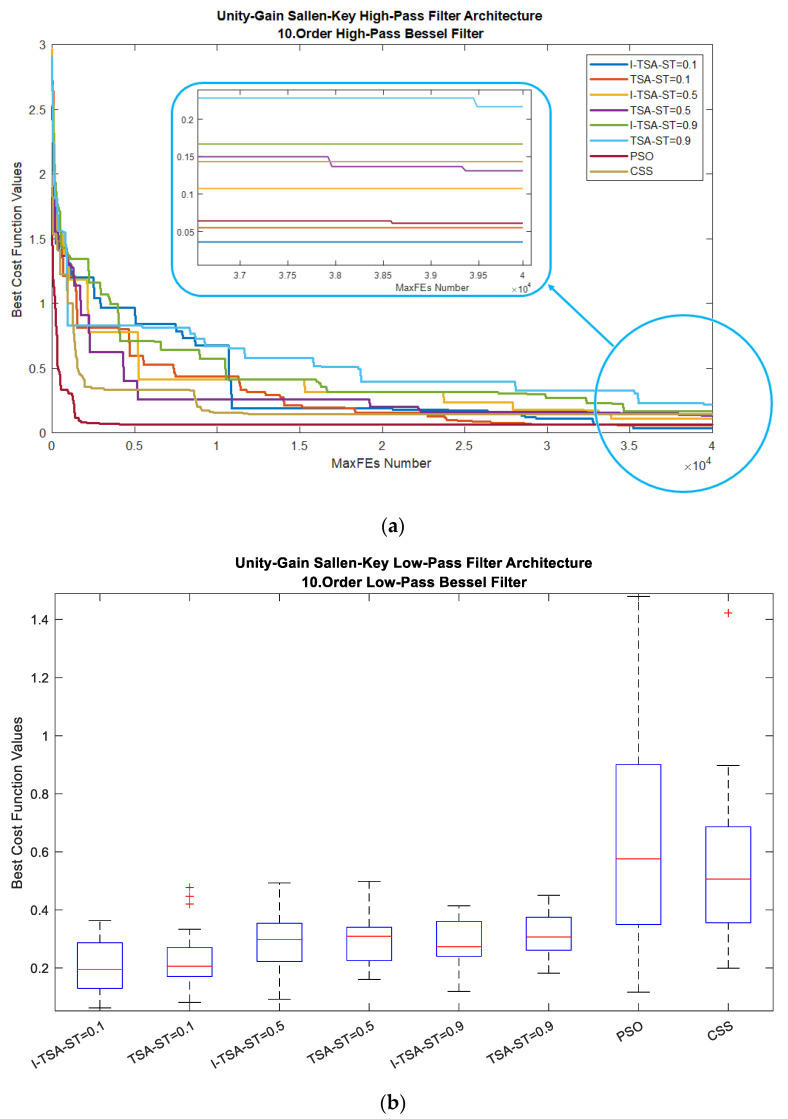

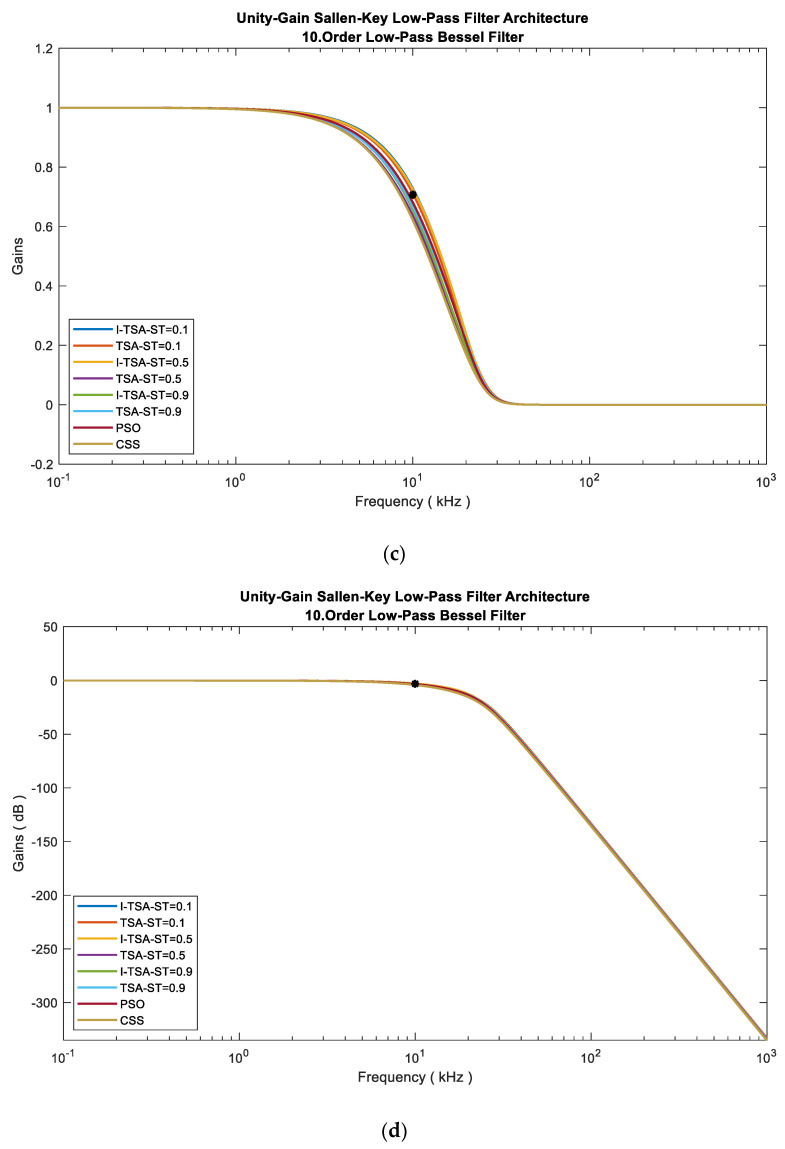
Plots for HPAF in the BF: (**a**) convergence graphs of I-TSA, TSA, PSO and CSS; (**b**) box plots of I-TSA, TSA, PSO and CSS; (**c**) frequency and gain graphs of I-TSA, TSA, PSO and CSS; (**d**) frequency and gain (dB) graphs of I-TSA, TSA, PSO and CSS.

When analyzed according to all algorithms, the graphs in Figure 9 are obtained. In Figure 9a, it is seen in the MaxFEs graph that when the ST value is 0.1, I-TSA achieves a more stable convergence and reaches the minimum error value. The box plots obtained from here are given in Figure 9b. In addition, the gain and gain (dB) graphs based on the minimum error value according to the ST values are given in Figure 9c,d, respectively.

## 6. Conclusions

The TSA method has become an alternative method for many real world problems. In this study, the I-TSA method, which is constructed by improving the TSA, is used for the first time in the literature to estimate the parameters of Butterworth and Bessel filters. For this study, I-TSA and TSA methods were analyzed for three different values of the ST parameter. These values are 0.1, 0.5 and 0.9, respectively. In the LPAF problem for Butterworth filter design, the I-TSA outperforms both the basic TSA and other algorithms with the least error value when the ST value of the I-TSA is 0.1. At the same time, in the HPAF problem, the least error value was obtained when the ST value of the I-TSA was 0.1. For Bessel filter design, in both the LPAF and HPAF problems, the I-TSA was more successful than other algorithms by obtaining the least error value when the ST value was 0.1. The success of the I-TSA was analyzed according to the ST values, and it was concluded that it achieved the best result at a value of 0.1. In addition, convergence graphs, box plots and gain graphs were drawn for all ST values in the I-TSA method. When these graphs are analyzed, it is seen that the I-TSA converges more successfully and produces more stable results than other algorithms when the ST value is 0.1 in all problems. As a result, the performance of the I-TSA is improved by contributing to the good results of the proposed OBL method for the TSA in the filter design problem.

In future studies, it is planned to use different plant-based algorithms in the filter design problem. It is also recommended to use the proposed I-TSA in different problems.

## Figures and Tables

**Table 1:** E24 series resistance and capacitors values.

1	1.1	1.2	1.3	1.5	1.6	1.8	2	2.2	2.4	2.7	3
3.3	3.6	3.9	4.3	4.7	5.1	5.6	6.2	6.8	7.5	8.2	9.1

**Table 2:** The best component values obtained for LPAF in the BWF.

Algorithm	ST Value	Component	S1	S2	S3	S4	S5
I-TSA	0.1	R_1_	1.6	1.5	6.8	1.5	3.0
		R_2_	1.6	2.2	3.3	1.8	2.0
		C_1_	10.0	7.5	2.2	4.3	1.0
		C_2_	10.0	10.0	5.1	22.0	43.0
I-TSA	0.5	R_1_	1.8	3.9	3.3	3.0	1.3
		R_2_	1.5	1.2	1.8	4.3	1.0
		C_1_	10.0	5.6	4.3	2.0	2.2
		C_2_	10.0	10.0	10.0	10.0	91.0
I-TSA	0.9	R_1_	1.0	3.3	4.3	5.1	2.0
		R_2_	1.2	7.5	6.8	3.9	4.7
		C_1_	15.0	2.7	2.0	1.6	0.8
		C_2_	15.0	3.6	4.3	8.2	36.0

**Table 3:** Parameter values obtained for LPAF in the BWF.

			S1	S2	S3	S4	S5
	Target	FSF	1.0000	1.0000	1.0000	1.0000	1.0000
		Q	0.5062	0.5612	0.7071	1.1013	3.1969
Algorithm	ST Value						
I-TSA	0.1	FSF	0.994718	1.011655	1.003026	0.995847	0.990855
		Q	0.500000	0.566924	0.714108	1.126277	3.212476
	0.5	FSF	0.968586	0.983112	0.995847	0.990855	0.986544
		Q	0.497930	0.566838	0.728767	1.100163	3.188256
	0.9	FSF	0.968586	1.026123	1.003650	0.985226	0.999020
		Q	0.497930	0.531904	0.714307	1.121816	3.170368

**Table 4:** Error value obtained for LPAF in the BWF.

Algorithm	ST Value	Best	Mean	Worst	Std. Dev.
I-TSA	0.1	**0.046585**	**0.212725**	0.413537	0.094703
	0.5	0.067909	0.291463	0.494240	0.100709
	0.9	0.091301	0.286216	0.448817	0.080759
TSA	0.1	0.075820	0.224486	0.386590	0.089920
	0.5	0.145739	0.313718	0.472144	0.085195
	0.9	0.150140	0.274506	0.423986	0.067385
PSO	-	0.110755	0.527483	0.965142	0.269997
CSS	-	0.081702	0.378726	0.992566	0.236921

**Table 5:** The best component values obtained for HPAF in the BWF.

Algorithm	ST Value	Component	S1	S2	S3	S4	S5
I-TSA	0.1	R_1_	3.9	6.2	8.2	10.0	18.0
		R_2_	2.7	4.7	2.7	2.0	0.4
		C_1_	8.2	2.4	1.8	3.9	10.0
		C_2_	3.0	3.6	6.8	3.3	3.9
I-TSA	0.5	R_1_	16.0	2.7	10.0	5.1	30.0
		R_2_	15.0	2.0	4.7	1.0	0.8
		C_1_	1.0	6.2	1.8	8.2	3.0
		C_2_	1.0	7.5	3.0	6.2	3.3
I-TSA	0.9	R_1_	2.0	4.3	10.0	24.0	51.0
		R_2_	1.8	3.0	4.7	3.6	1.0
		C_1_	6.8	3.6	1.8	1.0	4.7
		C_2_	10.0	5.6	3.0	3.0	1.0

**Table 6:** Parameter values obtained for HPAF in the BWF.

			S1	S2	S3	S4	S5
	Target	FSF	1.0000	1.0000	1.0000	1.0000	1.0000
		Q	0.5062	0.5612	0.7071	1.1013	3.1969
Algorithm	ST Value						
I-TSA	0.1	FSF	1.011257	0.996969	1.034331	1.008055	0.998849
		Q	0.532231	0.562668	0.708953	1.114145	3.176892
	0.5	FSF	0.973387	0.995642	1.000981	1.011737	0.980539
		Q	0.516398	0.578326	0.706166	1.118215	3.020861
	0.9	FSF	0.983073	1.013258	1.000981	1.011573	0.972778
		Q	0.517397	0.584293	0.706166	1.118034	2.716184

**Table 7:** Error value obtained for HPAF in the BWF.

Algorithm	ST Value	Best	Mean	Worst	Std. Dev.
I-TSA	0.1	**0.066204**	**0.223354**	0.405234	0.095903
	0.5	0.092779	0.305070	0.510234	0.105158
	0.9	0.150058	0.298889	0.452366	0.070469
TSA	0.1	0.084627	0.226826	0.499136	0.106131
	0.5	0.171201	0.319772	0.463289	0.075065
	0.9	0.208470	0.331249	0.475878	0.079518
PSO	-	0.079577	0.586805	1.436803	0.294510
CSS	-	0.127601	0.445885	1.334431	0.241027

**Table 8:** The best component values obtained for LPAF in the BF.

Algorithm	ST Value	Component	S1	S2	S3	S4	S5
I-TSA	0.1	R_1_	3.9	2.2	1.5	1.2	10.0
		R_2_	3.3	1.0	3.0	1.2	3.9
		C_1_	2.2	4.7	2.7	3.6	0.3
		C_2_	2.4	6.2	4.7	10.0	3.3
I-TSA	0.5	R_1_	9.1	4.7	3.9	7.5	3.3
		R_2_	7.5	10.0	6.8	3.0	5.1
		C_1_	1.0	1.0	1.1	0.8	0.6
		C_2_	1.0	1.3	2.0	2.7	4.7
I-TSA	0.9	R_1_	6.2	2.2	10.0	1.0	1.3
		R_2_	10.0	1.0	3.6	1.0	1.0
		C_1_	1.0	5.1	0.9	4.7	2.0
		C_2_	1.0	6.2	1.8	11.0	16.0

**Table 9:** Parameter values obtained for LPAF in the BF.

			S1	S2	S3	S4	S5
	Target	FSF	1.0000	1.0000	1.0000	1.0000	1.0000
		Q	0.5062	0.5612	0.7071	1.1013	3.1969
Algorithm	ST Value						
I-TSA	0.1	FSF	1.930696	1.987760	2.106120	2.210485	2.442159
		Q	0.520416	0.532364	0.621958	0.833333	1.420749
	0.5	FSF	1.926499	2.036102	2.083637	2.254966	2.391306
		Q	0.497672	0.531745	0.648966	0.819741	1.414874
	0.9	FSF	2.021270	1.908217	2.072583	2.213476	2.467593
		Q	0.486050	0.511060	0.620480	0.764922	1.402132

**Table 10:** Error value obtained for LPAF in the BF.

Algorithm	ST Value	Best	Mean	Worst	Std. Dev.
I-TSA	0.1	**0.062378**	**0.208454**	0.363140	0.092668
	0.5	0.092212	0.301908	0.492980	0.100669
	0.9	0.119140	0.289782	0.414542	0.073533
TSA	0.1	0.081274	0.227481	0.477295	0.097471
	0.5	0.160654	0.296257	0.498181	0.086586
	0.9	0.181832	0.310810	0.450243	0.072830
PSO	-	0.116503	0.645454	1.478782	0.330566
CSS	-	0.199537	0.541564	1.421876	0.264927

**Table 11:** The best component values obtained for HPAF in the BF.

Algorithm	ST Value	Component	S1	S2	S3	S4	S5
I-TSA	0.1	R_1_	16.0	12.0	12.0	16.0	22.0
		R_2_	10.0	6.8	7.5	4.7	2.7
		C_1_	1.2	1.8	4.3	2.4	4.3
		C_2_	5.1	6.8	2.7	6.8	6.2
I-TSA	0.5	R_1_	4.3	15.0	12.0	10.0	18.0
		R_2_	3.9	9.1	4.7	3.0	2.0
		C_1_	6.2	5.1	2.0	10.0	10.0
		C_2_	10.0	1.5	9.1	3.6	4.3
I-TSA	0.9	R_1_	10.0	4.3	10.0	39.0	18.0
		R_2_	11.0	3.6	5.6	13.0	2.4
		C_1_	2.4	8.2	2.7	2.0	6.2
		C_2_	3.6	8.2	7.5	1.2	5.1

**Table 12:** Parameter values obtained for HPAF in the BF.

			S1	S2	S3	S4	S5
	Target	FSF	1.0000	1.0000	1.0000	1.0000	1.0000
		Q	0.5062	0.5612	0.7071	1.1013	3.1969
Algorithm	ST Value						
I-TSA	0.1	FSF	1.966145	1.985709	2.031034	2.201146	2.500364
		Q	0.496701	0.540416	0.615713	0.810183	1.403687
	0.5	FSF	2.026013	2.030378	2.013053	2.064865	2.472096
		Q	0.510367	0.538036	0.614122	0.805474	1.375686
	0.9	FSF	1.937015	2.027119	2.115857	2.191742	2.322216
		Q	0.467099	0.546453	0.589547	0.838525	1.362803

**Table 13:** Error value obtained for HPAF in the BF.

Algorithm	ST Value	Best	Mean	Worst	Std. Dev.
I-TSA	0.1	**0.036035**	**0.190635**	0.353110	0.076729
	0.5	0.107378	0.332173	0.620112	0.124932
	0.9	0.166924	0.337793	0.522845	0.084933
TSA	0.1	0.054876	0.214660	0.513616	0.100502
	0.5	0.130932	0.323992	0.527150	0.097837
	0.9	0.216784	0.366686	0.492966	0.082912
PSO	-	0.061011	0.682382	1.360255	0.360903
CSS	-	0.143429	0.580740	1.075118	0.242949

## Data Availability

No new data were created or analyzed in this study. Data sharing is not applicable to this article.

## References

[1] Beşkirli A., Özdemir D., Temurtaş H. (2020). A comparison of modified tree–seed algorithm for high-dimensional numerical functions. Neural Comput. Appl..

[2] Beşkirli A., Temurtaş H., Özdemir D. (2020). Determination with linear form of Turkey’s energy demand forecasting by the tree seed algorithm and the modified tree seed algorithm. Adv. Electr. Comput. Eng..

[3] Beşkirli A., Dağ İ. (2020). A new binary variant with transfer functions of Harris Hawks Optimization for binary wind turbine micrositing. Energy Rep..

[4] Beşkirli A., Beşkirli M., Haklı H., Uğuz H. (2018). Comparing energy demand estimation using artificial algae algorithm: The case of Turkey. J. Clean Energy Technol..

[5] Wolpert D.H., Macready W.G. (1997). No free lunch theorems for optimization. IEEE Trans. Evol. Comput..

[6] Tefek M.F., Uğuz H., Güçyetmez M. (2019). A new hybrid gravitational search–teaching–learning-based optimization method for energy demand estimation of Turkey. Neural Comput. Appl..

[7] Tefek M.F. (2021). Artificial bee colony algorithm based on a new local search approach for parameter estimation of photovoltaic systems. J. Comput. Electron..

[8] Tefek M.F., Arslan M. (2022). Highway accident number estimation in Turkey with Jaya algorithm. Neural Comput. Appl..

[9] Jiang M., Yang Z., Gan Z. (2007). Optimal Components Selection for Analog Active Filters Using Clonal Selection Algorithms. Advanced Intelligent Computing Theories and Applications. With Aspects of Theoretical and Methodological Issues.

[10] Shakoor A., Abbas S., Abbas Z. (2019). Optimization of the Design Parameters of Low Pass Filter Using Genetic Algorithm. Univ. Wah J. Sci. Technol..

[11] De B.P., Kar R., Mandal D., Ghoshal S.P. (2015). Optimal selection of components value for analog active filter design using simplex particle swarm optimization. Int. J. Mach. Learn. Cybern..

[12] Temurtaş H. (2020). The estimation of low and high-pass active filter parameters with opposite charged system search algorithm. Expert Syst. Appl..

[13] Doğan B., Ölmez T. (2015). Vortex search algorithm for the analog active filter component selection problem. AEU-Int. J. Electron. Commun..

[14] Durmuş B., Temurtaş H., Özyön S. (2020). The design of multiple feedback topology Chebyshev low-pass active filter with average differential evolution algorithm. Neural Comput. Appl..

[15] Vural R.A., Bozkurt U., Yildirim T. (2013). Analog active filter component selection with nature inspired metaheuristics. AEU-Int. J. Electron. Commun..

[16] Beşkirli A., Dağ İ. (2023). Parameter extraction for photovoltaic models with tree seed algorithm. Energy Rep..

[17] Boumediene Ghaouti G., Meftah B. (2023). An Optimized Clustering Approach using Tree Seed Algorithm for the Brain MRI Images Segmentation. Intel. Artif..

[18] More S. (2023). Image Constraint Technique Used by Bio-Inspired Tsa Optimized Algorithm for Large Memory Management. SSRN.

[19] Beşkirli A., Dağ İ. (2022). An efficient tree seed inspired algorithm for parameter estimation of Photovoltaic models. Energy Rep..

[20] Mandal B., Chatterjee S., Roy P., Mukherjee I. (2023). A novel evolutionary algorithm named oppositional based chaotic tree seed algorithm (OCTSA) applied for energy cost minimization in hybrid microgrid system for different locations in India. Res. Sq..

[21] Venkatasubramanian S. Optimal Cluster head selection-based Hybrid Moth Search Algorithm with Tree Seed algorithm for multipath routing in WSN. Proceedings of the 2023 International Conference on Networking and Communications (ICNWC).

[22] Liu J., Hou Y., Li Y., Zhou H. (2023). A multi-strategy improved tree–seed algorithm for numerical optimization and engineering optimization problems. Sci. Rep..

[23] Sharshir S.S., Abd Elaziz M., Elsheikh A. (2023). Augmentation and prediction of wick solar still productivity using artificial neural network integrated with tree–seed algorithm. Int. J. Environ. Sci. Technol..

[24] Jiang J., Yang X., Li M., Chen T. (2023). ATSA: An Adaptive Tree Seed Algorithm based on double-layer framework with tree migration and seed intelligent generation. Knowl.-Based Syst..

[25] Liu J., Hou Y., Li Y., Zhou H. (2024). Advanced strategies on update mechanism of tree-seed algorithm for function optimization and engineering design problems. Expert Syst. Appl..

[26] Kiran M.S. (2015). TSA: Tree-seed algorithm for continuous optimization. Expert Syst. Appl..

[27] Aslan M.F., Sabanci K., Ropelewska E. (2022). A new approach to COVID-19 detection: An ANN proposal optimized through tree-seed algorithm. Symmetry.

[28] Jiang J., Meng X., Qian L., Wang H. (2022). Enhance tree-seed algorithm using hierarchy mechanism for constrained optimization problems. Expert Syst. Appl..

[29] Gharehchopogh F.S. (2022). Advances in Tree Seed Algorithm: A Comprehensive Survey. Arch. Comput. Methods Eng..

[30] Kiran M.S., Hakli H. (2021). A tree–seed algorithm based on intelligent search mechanisms for continuous optimization. Appl. Soft Comput..

[31] Chen X., Przystupa K., Ye Z., Chen F., Wang C., Liu J., Gao R., Wei M., Kochan O. (2022). Forecasting short-term electric load using extreme learning machine with improved tree seed algorithm based on Lévy flight. Eksploat. I Niezawodn..

[32] Tizhoosh H.R. Opposition-based learning: A new scheme for machine intelligence. Proceedings of the International Conference on Computational Intelligence for Modelling, Control and Automation and International Conference on Intelligent Agents, Web Technologies and Internet Commerce (CIMCA-IAWTIC′06).

[33] Wang H., Wu Z., Rahnamayan S., Liu Y., Ventresca M. (2011). Enhancing particle swarm optimization using generalized opposition-based learning. Inf. Sci..

[34] Gift S.J.G., Maundy B., Gift S.J.G., Maundy B. (2022). Active Filters. Electronic Circuit Design and Application.

[35] Beşkirli M., Egi Y. (2023). An efficient hybrid-based charged system search algorithm for active filter design. Neural Comput. Appl..

[36] Karki J. (2000). Active low-pass filter design. Tex. Instrum. Appl. Rep..

[37] Sallen R.P., Key E.L. (1955). A practical method of designing RC active filters. IRE Trans. Circuit Theory.

[38] Pactitis S. (2018). Active Filters: Theory and Design.

